# The Targeted Deletion of Genes Responsible for Expression of the Mth60 Fimbriae Leads to Loss of Cell-Cell Connections in Methanothermobacter thermautotrophicus ΔH

**DOI:** 10.1128/aem.00575-23

**Published:** 2023-06-13

**Authors:** Christian Fink, Gines Martinez-Cano, Jeremiah Shuster, Aurora Panzera, Kim E. Rennhack, Nils Rohbohm, Largus T. Angenent, Bastian Molitor

**Affiliations:** a Environmental Biotechnology Group, Department of Geosciences, University of Tübingen, Tübingen, Germany; b Tübingen Structural Microscopy, University of Tübingen, Tübingen, Germany; c BioOptics Facility, Max Planck Institute for Biology Tübingen, Tübingen, Germany; d Cluster of Excellence–Controlling Microbes to Fight Infections, University of Tübingen, Tübingen, Germany; e AG Angenent, Max Planck Institute for Biology Tübingen, Tübingen, Germany; f Department of Biological and Chemical Engineering, Aarhus University, Aarhus C, Denmark; g The Novo Nordisk Foundation CO_2_ Research Center (CORC), Aarhus University, Aarhus C, Denmark; Kyoto University

**Keywords:** archaea, genetics, *Methanothermobacter*, Mth60 fimbriae, gene deletion, adherence

## Abstract

This study is a continuation by the Environmental Biotechnology Group of the University of Tübingen in memoriam to Reinhard Wirth, who initiated the work on Mth60 fimbriae at the University of Regensburg. Growth in biofilms or biofilm-like structures is the prevailing lifestyle for most microbes in nature. The first crucial step to initiate biofilms is the adherence of microbes to biotic and abiotic surfaces. Therefore, it is crucial to elucidate the initial step of biofilm formation, which is generally established through cell-surface structures (i.e., cell appendages), such as fimbriae or pili, that adhere to biotic and abiotic surfaces. The Mth60 fimbriae of Methanothermobacter thermautotrophicus ΔH are one of only a few known archaeal cell appendages that do not assemble via the type IV pili assembly mechanism. Here, we report the constitutive expression of Mth60 fimbria-encoding genes from a shuttle-vector construct and the deletion of the Mth60 fimbria-encoding genes from the genomic DNA of *M. thermautotrophicus* ΔH. For this, we expanded our system for genetic modification of *M. thermautotrophicus* ΔH using an allelic-exchange method. While overexpression of the respective genes increased the number of Mth60 fimbriae, deletion of the Mth60 fimbria-encoding genes led to a loss of Mth60 fimbriae in planktonic cells of *M. thermautotrophicus* ΔH compared to the wild-type strain. This, either increased or decreased, number of Mth60 fimbriae correlated with a significant increase or decrease of biotic cell-cell connections in the respective *M. thermautotrophicus* ΔH strains compared to the wild-type strain.

**IMPORTANCE** *Methanothermobacter* spp. have been studied for the biochemistry of hydrogenotrophic methanogenesis for many years. However, a detailed investigation of certain aspects, such as regulatory processes, was impossible due to the lack of genetic tools. Here, we amend our genetic toolbox for *M. thermautotrophicus* ΔH with an allelic exchange method. We report the deletion of genes that encode the Mth60 fimbriae. Our findings provide the first genetic evidence of whether the expression of these genes underlies regulation and reveal a role of the Mth60 fimbriae in the formation of cell-cell connections of *M. thermautotrophicus* ΔH.

## INTRODUCTION

Microbial biofilm formation, maintenance, and dispersion in various habitats have been investigated in numerous studies (1–3). Many studies elucidated biofilm formation for bacteria, especially for clinically relevant pathogenic species (3–6). However, knowledge about archaeal biofilm formation remains in an early stage (7, 8). Archaea are found in extreme habitats with respect to pH, temperature, or salinity, as well as under moderate conditions such as seawater, the human gut, and rice paddy fields. Thus, archaea have evolved a wide range of strategies to colonize this extensive variety of habitats (9). In general, forming a biofilm in new habitats is performed through cell-surface molecules and structures, enabling microbes to attach and adhere to a surface (10, 11). One possibility is adherence via extracellular polymeric substances (12). However, this was more frequently described to be important in a later stage of colonization and not for the initial attachment (3, 12). For archaea, this initial attachment to surfaces mostly relies on archaeal cell appendages, such as archaella, pili, fimbriae, and other specialized archaeal cell appendages (13, 14). Typically, archaella differ from all other cell appendages in two ways: (i) the diameter, which is 10 to 15 nm, compared to ~5 nm for fimbriae and pili, and (ii) the ability to rotate, and therefore enable directed motility of the microbe (13). It was shown that several archaellum structures allow for adherence to surfaces (14–16). Archaella and most cell appendages described for archaea, so far, are assembled via the type IV pili assembly mechanism (17, 18). However, some archaeal cell appendages most likely assemble by mechanisms different from the type IV pili assembly mechanism, such as bundling pili of Pyrobaculum calidifontis, archaeal cannulae of Pyrodictium abysi, hami from Altiarchaeum hamiconexum, threads of Sulfolobus acidocaldarius, conjugative pili of Aeropyrum pernix and *P. calidifontis*, and Mth60 fimbriae from *M. thermautotrophicus* ΔH (19–25). These archaeal cell appendages allow adherence to abiotic surfaces and biotic adherence between microbes but do not confer motility to the microbe.

Here, we focused on the Mth60 fimbriae from *M. thermautotrophicus* ΔH. The Mth60 fimbriae were first described by Doddema et al. (26). They differ from archaella by their diameter and a length of up to 5 μm (13, 26). Planktonic wild-type *M. thermautotrophicus* ΔH cells contain between one and three Mth60 fimbriae. In contrast, cells that adhered to surfaces contained significantly higher numbers of Mth60 fimbriae per cell (20). *M. thermautotrophicus* ΔH was shown to adhere to several distinct surfaces, such as glass, carbon-coated gold, copper grids, and silicium wafers, via the Mth60 fimbriae (20). In addition to abiotic surfaces, biotic cell-cell connections with surface-adhered *M. thermautotrophicus* ΔH have been demonstrated (20).

The Mth60 fimbriae mainly consist of the major fimbrin protein Mth60, which is eponymous for the Mth60 fimbriae. The corresponding gene, *mth60*, is transcribed in two transcriptional units (i.e., operons), *mth58-mth60* and *mth60-mth61* (MTH_RS00275 to MTH_RS00285, MTH_RS00285 to MTH_RS00290). Therefore, the transcription levels of *mth60* are much higher than those of the surrounding genes in the two operons (27). Recombinant Mth60 protein, produced in Escherichia coli, led to auto-assembly of filamentous fimbria structures when incubated at 65°C in *M. thermautotrophicus* ΔH growth medium (20, 27, 28). This auto-assembly feature of recombinant Mth60 protein was patented for a potential application as heat-induced glue through the solidification of the Mth60 protein at elevated temperatures (28). Furthermore, the auto-assembly feature indicated a unique pilus assembly mechanism of Mth60 fimbriae, which is distinct from the type IV pilus assembly mechanism that was described for the majority of cell appendages studied to date in archaea (13). The functions of *mth58*, *mth59*, and *mth61*, which are the three genes that are cotranscribed with *mth60*, remain largely unknown. Auto-assembly tests of Mth59 together with Mth60 failed in assembling filamentous structures. However, additional bioinformatics modeling of the Mth59 protein structure indicated a potential chaperone function of Mth59 for Mth60 (27).

Biofilm formation of methanogenic archaea has been demonstrated in different habitats with various methanogenic species involved, such as (i) biofilm formation of Methanosphaera stadtmanae and Methanobrevibacter smithii in the human gut (29), (ii) syntrophic relationships of the sulfate-reducing bacteria Desulfovibrio vulgaris Hildenborough and Methanococcus maripaludis (30), and (iii) putative colonization of black smokers by Methanocaldococcus villosus (31). To further investigate the relevance of Mth60 fimbriae of the thermophilic methanogenic archaeon *M. thermautotrophicus* ΔH for biotic cell-cell connections (20), we expanded our genetic toolbox for *M. thermautotrophicus* ΔH (32) with suicide vectors for targeted gene deletion. This enabled us to delete the Mth60 fimbria-encoding operons (*mth58*-*mth60* and *mth60*-*mth61*) from the genomic DNA of *M. thermautotrophicus* ΔH using an allelic-exchange method. We further generated a strain of *M. thermautotrophicus* ΔH that contained a shuttle-vector construct for the constitutive expression of the Mth60 fimbria-encoding operons. We observed various phenotypes and significantly different numbers of Mth60 fimbriae per cell for the different strains. Thus, we could elucidate the intraspecies adherence ability of *M. thermautotrophicus* ΔH.

## RESULTS

### Suicide-vector constructs allow site-specific deletion of Mth60 fimbria-encoding operons in *M. thermautotrophicus* ΔH.

**FIG 1:**
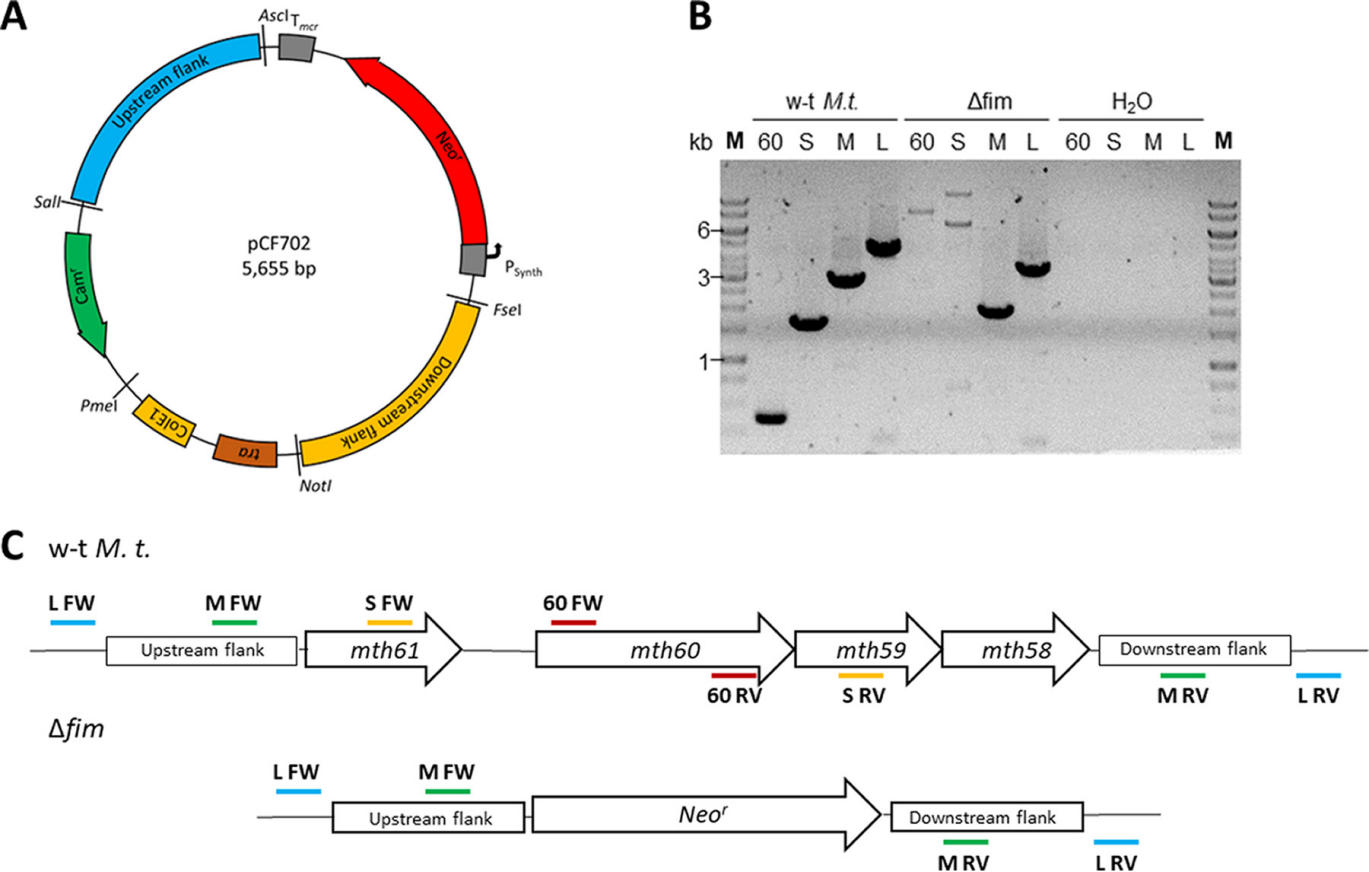
(A to C) Plasmid map of pCF702, a suicide-vector construct for deletion of the Mth60 fimbria-encoding operons (A) and the agarose gel of a corresponding PCR analysis (B), including a schematic depiction of the genomic region before (wild-type *M. thermautotrophicus* [w-t *M.t.*]) and after the deletion (Δfim) of *mth58-mth61* (C). (A) The suicide-vector construct pCF702 consists of five exchangeable modules flanked by unique restriction enzyme-recognition sites, as stated in parentheses below. The origin of replication ColEI for E. coli, including a *tra* region for mobilization during conjugation (NotI, PmeI), the antibiotic resistance against chloramphenicol (Cam^r^) as a selectable marker for E. coli (PmeI, SalI), and 1-kb upstream (SalI, AscI) and downstream homologous regions (FseI, NotI) for homologous recombination in *M. thermautotrophicus* ΔH are shown. A thermostable neomycin resistance gene (Neo^r^) with constitutive promoter P_Synth_ and terminator T*_mcr_* as a selectable marker for *M. thermautotrophicus* ΔH is located between the homologous flanking regions (AscI, FseI). (B) PCR analysis with four primer combinations to confirm the Mth60-fimbriae operon deletion. Two primer combinations amplify a fragment inside the Mth60 fimbria-encoding operons (60, S), and two primer combinations amplify a fragment outside the Mth60 fimbria-encoding operons (M, L). These combinations result in amplified fragments of reduced lengths since the Mth60 fimbria-encoding operons (2.8 kb) were substituted with Neo^r^ (1.2 kb). (C) The schematic depiction of the genomic region before and after the deletion of *mth58-61* shows the distribution of the individual genes *mth58* to *mth61* and the substitution with Neo^r^ after integration of the suicide-vector construct. The lines depicting primer pairs L (light blue), M (green), S (yellow), and 60 (dark red) correspond with the fragments in panel B. FW and RV represent the forward and reverse complements of the respective primer combinations. While fragments M and L appear in different lengths in both strains, S and 60 are present only in wild-type *M. thermautotrophicus* ΔH.

We chose suicide-vector constructs to substitute *mth58-mth61* (i.e., the Mth60 fimbria-encoding operons MTH_RS00275 to MTH_RS00290) with a positive selectable marker. Therefore, we created suicide-vector constructs with ~1-kb homologous flanking regions upstream and downstream of the Mth60 fimbria-encoding operons (Fig. 1). We placed unique restriction enzyme-recognition sites at the interfaces between the homologous flanking regions, which rarely/do not occur in the genome of *M. thermautotrophicus* ΔH. While SalI and NotI occur 137 and 7 times, respectively, on the genome of *M. thermautotrophicus* ΔH, AscI and FseI are not present at all. Using these restriction enzyme-recognition sites, we ensured that exchangeability with virtually all homologous flanking regions for *M. thermautotrophicus* ΔH genes is possible. This modularity facilitates the generation of future suicide-vector constructs. We implemented the restriction enzyme-recognition sites AscI and FseI at the selectable-marker interfaces. With that, it is also possible to directly implement selectable markers from the pMVS shuttle-vector design for *M. thermautotrophicus* ΔH into suicide-vector constructs (32). As the selectable marker, we used the thermostable neomycin resistance gene under the control of the P_Synth_ promoter (32–34). The commonly used T*_mcr_*_(_*_M.v._*_)_ sequence served as the terminator (35).

We performed the conjugations of *M. thermautotrophicus* ΔH with the suicide-vector construct as described before (32). For the first enrichment step in selective liquid medium, we did not recognize any differences in terms of efficiency compared to conjugation experiments with shuttle-vector constructs. However, for this initial enrichment step in selective liquid medium and for plating of *M. thermautotrophicus* ΔH deletion mutants, we applied 100 μg/mL of neomycin instead of 250 μg/mL, which we used for all subsequent selective liquid medium incubations. With that, we ensured the generation of individual clonal populations on selective solidified medium plates because no clonal population appeared on plates with the higher antibiotic concentration.

While screening for a clean *M. thermautotrophicus* ΔH strain with a deletion of the Mth60 fimbria-encoding operons, we continuously found wild-type signals in the PCR analysis in addition to the correct signal for double-homologous recombination events (see Fig. S2 in the supplemental material). Nanopore sequencing of one of these cultures with mixed PCR signals revealed the coexistence of single- and double-homologous recombination events of the suicide vector with genomic DNA of *M. thermautotrophicus* ΔH (Fig. S4). In contrast, wild-type *M. thermautotrophicus* ΔH nanopore sequencing reads did not align with the neomycin resistance gene (Fig. S3). After an additional screening step with four individual clonal populations, we isolated an *M. thermautotrophicus* ΔH Δ*mth58-61*:Neo^r^ mutant without a wild-type genomic DNA background (Fig. 1B). We confirmed the absence of wild-type *mth58-61* with the help of two specific primer combinations, which only bind to the genomic DNA of wild-type *M. thermautotrophicus* ΔH (Fig. 1B and C; S, 60). Furthermore, with two additional specific primer pairs that result in different fragment lengths of the PCR product, we confirmed the substitution of the Mth60 fimbria-encoding operons with the neomycin selectable marker because the Neo^r^ gene is smaller than the four genes *mth58* to *mth61* (Fig. 1C, M, L). The latter primer combinations would result in two PCR fragments if wild-type *M. thermautotrophicus* ΔH genomic DNA background was still present. Thus, the uniformity of the genotype, and therefore the purity of the *M. thermautotrophicus* ΔH Δ*mth58-61*:Neo^r^ strain was confirmed (Fig. 1B).

In addition to the Mth60 fimbria deletion strain of *M. thermautotrophicus* ΔH, we generated an *M. thermautotrophicus* ΔH strain (*M. thermautotrophicus* ΔH pMVS1111A:P*_hmtB_*-*mth58-61*) that constitutively expressed the Mth60 fimbria-encoding operons. For this, we exchanged the gene of interest module of the pMVS1111A:P_Synth_-*bgaB* shuttle vector with the *mth58* to *mth61* genes under the control of the P*_hmtB_* promoter, which substituted the putative promoter region that is located upstream of *mth61* (Fig. S1A) (32). After the conjugation of wild-type *M. thermautotrophicus* ΔH with the shuttle-vector construct for constitutive expression, we confirmed the maintenance of the construct after three and four transfers of the culture with a specific primer combination for the origin of replication module (Fig. S1B).

### Constitutive expression of Mth60 fimbria-encoding operons results in an increase and deletion results in a loss of visualizable Mth60 fimbriae compared to wild-type *M. thermautotrophicus* ΔH.

The *M. thermautotrophicus* ΔH mutant strains for constitutive expression and with the deletion of Mth60 fimbriae-encoding operons allowed us to compare the resulting phenotypes to wild-type *M. thermautotrophicus* ΔH and with each other. To analyze the phenotypes, we chose two distinct microscopical methods to visualize Mth60 fimbriae. First, we used immunofluorescence staining with confocal light microscopy, and second, scanning electron microscopy of native *M. thermautotrophicus* ΔH (mutant) strain samples.

**FIG 2:**
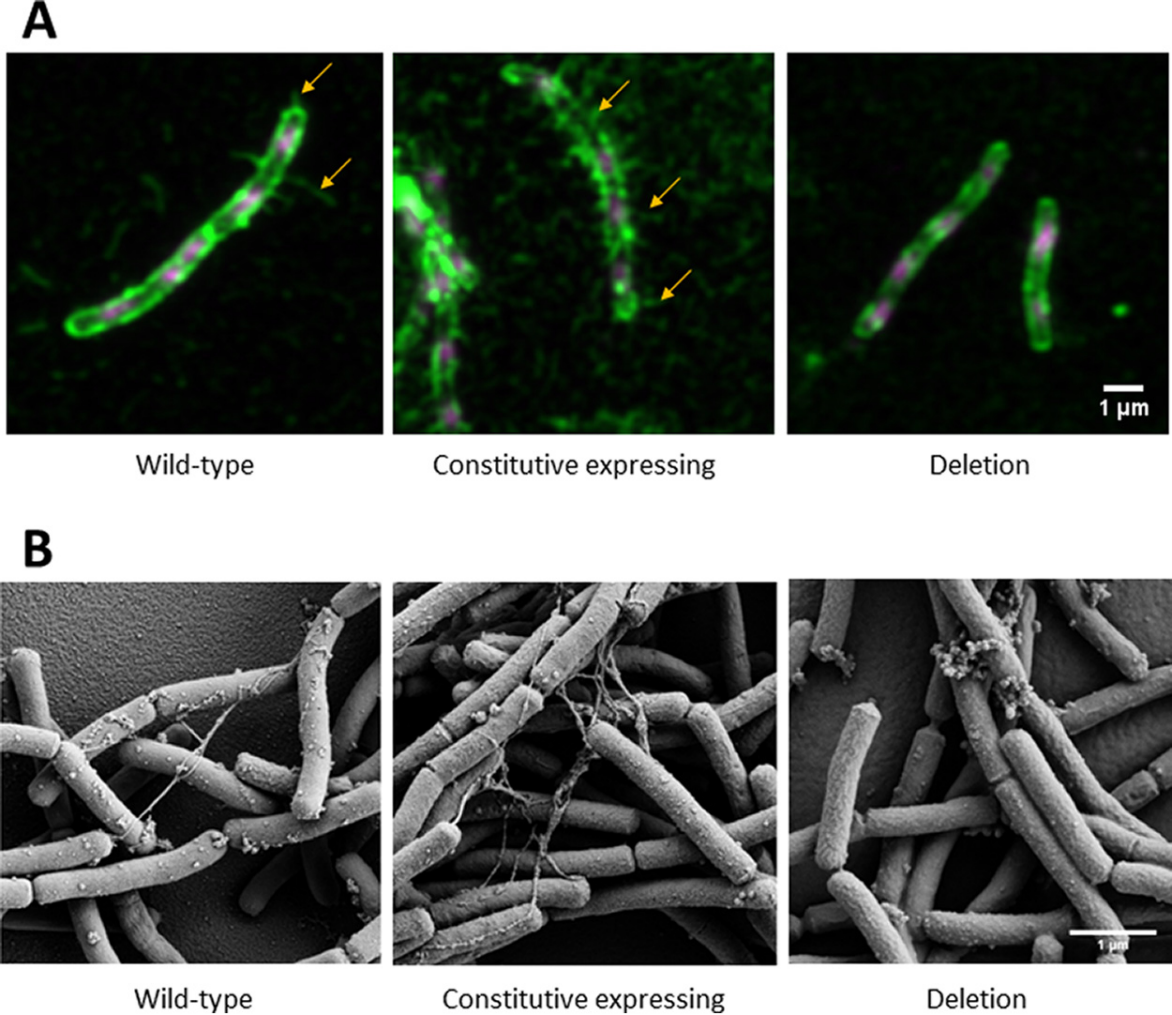
(A and B) Two-channel maximum intensity z-projection of representative Airyscan-processed z-stacks of immunofluorescence-stained *M. thermautotrophicus* ΔH strains (A) and scanning electron micrographs of the same three untreated *M. thermautotrophicus* ΔH strains (B). (A) For the Airyscan process z-stacks, DAPI staining is shown in magenta. The Alexa Fluor 488 conjugated antibody, which is attached to the primary anti-Mth60-fimbria antibody, is depicted in green. Orange arrows indicate Mth60-fimbria structures, and the 1-μm scale bar represents the size for all pictures because the magnification is the same. Three *M. thermautotrophicus* strains were analyzed: *M. thermautotrophicus* ΔH wild-type (wild-type), *M. thermautotrophicus* ΔH containing a shuttle vector for constitutive expression of the Mth60 fimbria-encoding operons (constitutive expression), and *M. thermautotrophicus* ΔH with a deletion of the Mth60 fimbria-encoding operons (deletion). (B) The scale bar indicates 1 μm for all three images for the scanning electron micrographs. The same strains of *M. thermautotrophicus* ΔH as in panel A were analyzed and are depicted in the same order.

For immunofluorescence staining, we applied an anti-Mth60-fimbria antibody as the first antibody. The antibody was obtained using a fraction, which was highly enriched in Mth60 fimbriae (Christina Sarbu, University of Regensburg) (27). The anti-Mth60-fimbria antibody does bind to Mth60 fimbriae. However, it was also shown to bind to other cell-membrane components. This resulted in immunofluorescence staining of the entire *M. thermautotrophicus* ΔH cells, in addition to the Mth60 fimbriae (Fig. 2; larger field of view in Fig. S5). The anti-Mth60-fimbria antibody most likely recognizes surface glycans, therefore staining most cell surface and surface structures. To further corroborate the binding capacity of the anti-Mth60-fimbria antibody to Mth60 fimbriae, we performed a Western blot analysis of crude extracts of the three *M. thermautotrophicus* ΔH strains (wild-type, constitutive expression, and deletion strains). We performed an SDS-PAGE analysis of the cytoplasmic and insoluble fractions of the three strains. We found that the 16-kDa protein Mth60 was not present in the Mth60-fimbria deletion strain, while the signal for it appeared stronger in the constitutive Mth60-fimbria expression strain than in the wild-type (Fig. S7A, black box). Thus, we provided further evidence for the absence of Mth60 in the deletion strain with the antibody reaction of the anti-Mth60-fimbria antibody against Mth60 (Fig. S7B). However, the antibody in our analysis also generated nonspecific reactions with proteins in the crude cell extract (Fig. S7B).

We passively attached planktonic wild-type *M. thermautotrophicus* ΔH from liquid medium on poly-lysine glass slides. After immunofluorescence staining, we found one up to a few stained Mth60 fimbriae per planktonic wild-type *M. thermautotrophicus* ΔH cell (Fig. 2A). Similar numbers of Mth60 fimbriae for planktonic wild-type *M. thermautotrophicus* ΔH were also described in Thoma et al. (20). Thus, we analyzed specimens of the *M. thermautotrophicus* ΔH pMVS1111A:P*_hmtB_*-*mth58-61* that constitutively expressed Mth60 fimbriae. We found several Mth60 fimbriae per cell, which vastly exceeded the numbers in stained wild-type *M. thermautotrophicus* ΔH cells (Fig. 2A). On the other hand, we compared the *M. thermautotrophicus* ΔH Δ*mth58-61*::Neo^r^ strain to the wild-type strain with the same immunofluorescence staining procedure. We found that specimens of the *M. thermautotrophicus* ΔH Δ*mth58-61*::Neo^r^ strain contained the stained cell wall but did not show any Mth60 fimbriae (Fig. 2A). Additionally, we frequently observed detached/solitary Mth60 fimbriae in the constitutive expression strain and low numbers for the wild-type strain but never in the Mth60-fimbria deletion strain.

The immunofluorescence staining enabled us to visualize various numbers of Mth60 fimbriae for the different *M. thermautotrophicus* ΔH strains (wild-type, constitutive expression, and deletion strain). However, we aimed for another layer of evidence to confirm the differences between the three strains and decided to employ scanning electron microscopy (SEM). To maintain a state that is closest to the physiological conditions in the serum bottles, we passively attached the planktonic *M. thermautotrophicus* ΔH cells to poly-lysine-coated SEM coverslips. This avoided centrifugation and, therefore, potential disruption of Mth60 fimbriae. The scanning electron microscopical analysis results of wild-type *M. thermautotrophicus* ΔH aligned with the observations from immunofluorescence staining and revealed, in general, one up to a few Mth60 fimbriae per *M. thermautotrophicus* ΔH wild-type cell (Fig. 2B, wild type). In the constitutive expression strain, the Mth60 fimbriae appeared to be more frequent than in specimens of wild-type *M. thermautotrophicus* ΔH (Fig. 2B, constitutive expressing). These Mth60 fimbriae were visible as filaments connecting different cells (Fig. 2B, constitutive expressing). However, the difference did not appear as strong as the immunofluorescence staining procedure indicated.

Furthermore, it cannot be entirely excluded that the high number of cell appendage structures was a secondary effect of cellular processes induced by the constitutive expression of the Mth60-fimbriae operons. We did not observe Mth60 fimbriae that connect cells with each other in the Mth60-fimbria deletion strain, such as we did for wild-type *M. thermautotrophicus* ΔH and the constitutive expression strain specimens (Fig. 2B, deletion). In all specimens, including in the Mth60-fimbria deletion strain, we found additional extracellular spherical structures attached to cells. These spherical structures did not connect cells with each other and did not resemble the filamentous structure of Mth60 fimbriae that we found only in wild-type *M. thermautotrophicus* ΔH and the constitutive expression strain.

### The number of Mth60 fimbriae significantly influences the intraspecies biotic interaction ability of *M. thermautotrophicus* ΔH.

**FIG 3:**
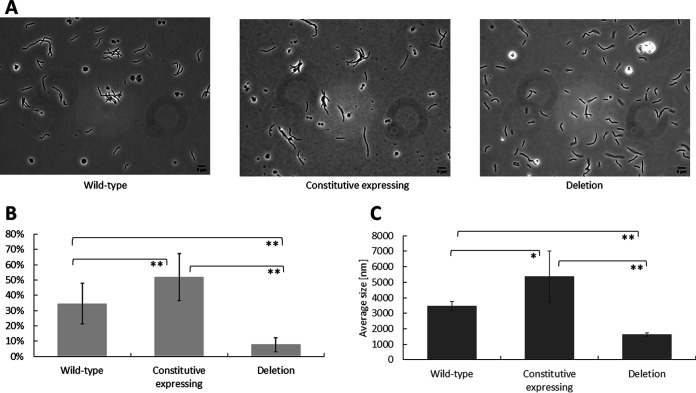
(A to C) Phase-contrast microscopy pictures of three *M. thermautotrophicus* ΔH strains (A) as representative pictures on which the analysis of the cell-cell connections was based (B); the average cell-cluster size was measured with a Zeta analyzer (C). (A) Representative phase-contrast microscopy pictures of the three analyzed strains, the wild-type, the constitutive Mth60-fimbria expression strain, and the Mth60 fimbria deletion strain of *M. thermautotrophicus* ΔH. (B) Comparison of the three *M. thermautotrophicus* ΔH strains mentioned above regarding the total number of microbial cells with the number of cells connected to another cell in percentage. Average (*N* = 3, *n* = 10) with error bars indicating the standard deviation. Significance was tested with ANOVA (*F* [102.6] > *F*_crit_ [3.2]), followed by individual Student’s *t* test (two-tailed) between two respective strains; *, significant difference (*P* < 0.05); **, highly significant difference (*P* < 0.01). (C) Results of Zeta analyzer analysis regarding average particle size in nm of the microbial cell clusters of the three *M. thermautotrophicus* ΔH strains depicted in panel A. The same statistical approach was followed as for panel B with ANOVA (*F* [21.5] > *F*_crit_ [3.4]), and asterisks reflect the same confidence intervals of the *P* value.

The experiments described above strongly indicated an increase of Mth60 fimbriae in the constitutive expression strain and a loss of Mth60 fimbriae in the deletion strain compared to wild-type *M. thermautotrophicus* ΔH. Thus, we hypothesized that these changes would be reflected in physiological differences between the strains. All three strains (wild-type, constitutive expression, and deletion strains) grew overnight to a final optical density at 600 nm (OD_600_) of around 0.3. However, we observed a considerable difference between the strains with phase-contrast microscopy without further treatment of the samples after harvesting in the early stationary growth phase. *M. thermautotrophicus* ΔH forms multicellular filaments (36) with approximately 3 to 5 cells per filament in our cultivation conditions. Those multicellular filaments are considered one planktonic cell in the following paragraph. Attachments between those multicellular filaments will be called “cellular clusters.” For wild-type *M. thermautotrophicus* ΔH, we found several cellular clusters but also cells that remained planktonic (Fig. 3A, wild type). For the constitutive expression strain, the ratio of cellular clusters to planktonic cells shifted toward cellular clusters (Fig. 3A, constitutive expressing). In contrast, for the deletion strain, it shifted toward planktonic cells (Fig. 3A, deletion). This finding prompted us to develop a method to define significant differences in the number of cellular clusters, and thus to determine a significant physiological difference between the three strains. Therefore, we collected a relevant number of phase-contrast microscopy images (*n* = 10, technical replicates) from biological replicates (*N* = 3) for each strain, resulting in 30 analyzed pictures per strain. Afterward, we counted (i) the total number of cells per image and (ii) the number of cells that had an immediate connection to another cell. It was crucial to dilute the cells to the same optical density (OD_600_, ~0.28) to gather comparable results. While in wild-type *M. thermautotrophicus* ΔH, 34.5% (±13%) of the cells showed a connection to another cell, this was 52% (±15%) in the constitutive expression strain. The Mth60-fimbria deletion strain showed only a remaining 7.5% (±4.5%) of cells that were connected to other cells. A statistical analysis of variance (ANOVA) of the ratio of the total number of cells to the number of connected cells indicated significant differences in cell-cell connections among the three strains (Fig. 3B). Further Student’s *t* test analyses between combinations of two strains revealed statistically significant differences between all combinations of the three strains (Fig. 3B).

In a second approach, we analyzed the average cellular-cluster sizes (diameter in nm) of the strains with a Zetasizer analysis. The analysis revealed results that were complementary to the phase-contrast microscopy approach. In wild-type *M. thermautotrophicus* ΔH, the average cellular-cluster size was 3,500 (±290) nm, while it was 5,400 (±1,600) nm in the constitutive expression strain and 1,600 (±110) nm in the deletion strain (Fig. 3C). According to ANOVA, the cellular-cluster size in nm differed significantly among the three strains. Student’s *t* tests between the strains resulted in significant differences between all combinations of the three strains (Fig. 3C).

## DISCUSSION

In this study, we reported the implementation of suicide-vector constructs for homologous recombination in *M. thermautotrophicus* ΔH to generate site-specific gene deletion mutants via allelic exchange with a positive selectable marker. With our expanded genetic tools, we elucidated the positive and negative influence of constitutive expression and deletion of the Mth60 fimbria-encoding operons on the *in-vivo* production of Mth60 fimbriae in *M. thermautotrophicus* ΔH. We demonstrated a correlation between the number of Mth60 fimbriae and the number of cell-cell connections with a constitutive Mth60-fimbria expression strain, wild-type strain, and Mth60-fimbria deletion strain of *M. thermautotrophicus* ΔH. We measured significantly lower numbers of cell-cell connections in *M. thermautotrophicus* ΔH strains with lower numbers of Mth60 fimbriae. Therefore, we demonstrated the importance of Mth60 fimbriae for establishing cell-cell connections, which is essential for initial biofilm formation.

The DNA-transfer protocol, and therefore the generation of deletion mutants of *M. thermautotrophicus* ΔH, was performed with the same procedure we had established before for shuttle-vector constructs (32). However, for the successful isolation of mutant strains, the concentration of neomycin as the antibiotic substance had to be lowered to 100 μg/mL instead of 250 μg/mL for the initial liquid enrichment and on solidified medium plates when a genomic alteration was introduced. It is known that cells can adapt the copy number of plasmids in response to higher antibiotic substance concentrations, which leads to higher resistance levels toward these antibiotic substances (37). It is further known that *M. thermautotrophicus* ΔH is always diploidic (38). Thus, we argue that the copy number of our shuttle vector is likely higher than two (as for the genome copies) or potentially can be increased with higher antibiotic substance concentrations. This would explain the higher neomycin resistance levels of shuttle-vector-containing *M. thermautotrophicus* ΔH compared to genome-altered *M. thermautotrophicus* ΔH mutant strains.

While isolating a clean *M. thermautotrophicus* ΔH strain with a deletion of the Mth60 fimbria-encoding operons, we obtained PCR signals and Nanopore sequencing reads for wild-type *M. thermautotrophicus* ΔH, single-homologous recombined, and double-homologous recombined mutant strains from the same colony sample. This was the case even after two steps that included isolating an individual clonal population and transferring it to liquid growth medium (Fig. S2). One possible explanation is the diploid character of *M. thermautotrophicus* ΔH, which might result in residual wild-type or single-homologous recombined alleles on the second chromosome (38). This could result in a heterozygous culture of *M. thermautotrophicus* ΔH, as it was shown to appear in heterozygous and many genome copy-containing Methanococcus maripaludis cultures (39). Another possible explanation is the characteristic of *M. thermautotrophicus* ΔH of forming multicellular filaments. This could result in different genotypes in one filament of multiple individual *M. thermautotrophicus* ΔH cells (36, 38). These observations of various genotypical PCR signals make it more laborious, but not impossible, to isolate clean deletion strains of *M. thermautotrophicus* ΔH with a homologous recombination-based methodology (Fig. 1B). To reduce the required screening efforts, tools for markerless mutagenesis or CRISPR/Cas should be implemented in the future, because they were shown to work in other methanogenic archaea already (40–43).

We performed immunofluorescence staining to visualize the Mth60 fimbriae with the Mth60-fimbria-deletion, the constitutive Mth60-fimbria-producing, and wild-type *M. thermautotrophicus* strains. The Mth60-fimbria antibodies that we used for immunofluorescence staining were generated from a native Mth60-fimbria preparation, which was purified through density gradient centrifugation. After our staining approach, we demonstrated that the entire cell wall was also stained, in addition to the Mth60 fimbriae, which resulted in the staining of the entire cell (Fig. 2A to C). One possible explanation is that cell wall components were purified in the same fraction of the density gradient centrifugation, resulting in a mixture of the polyclonal antibodies against several antigens. Another explanation is that the Mth60 fimbria antibody recognizes glycosylated epitopes of the major fimbrin Mth60 of the Mth60 fimbriae (20). In that case, the Mth60-fimbria antibody might also bind glycosylated cell wall components on the envelope of *M. thermautotrophicus* ΔH cells (44).

Thoma et al. (20) mentioned a difference in the number of Mth60 fimbriae in planktonic *M. thermautotrophicus* ΔH cells versus cells that were actively grown in the presence of a surface to which the cells adhered. While only 50% of planktonic cells contained few Mth60 fimbriae, cells that were adhered to surfaces contained large numbers of Mth60 fimbriae per microbial cell (20). This finding indicated regulation of the expression of the Mth60 fimbria-encoding operons. When we exchanged the putatively regulated promoter for the constitutive P*_hmtB_* promoter, fimbriae were identified in higher numbers for each planktonic *M. thermautotrophicus* ΔH cell (Fig. 2 and 3) (32). However, the regulatory mechanism of putative promoter regions of the Mth60 fimbria-encoding operons will need to be investigated further.

The Mth60-fimbria deletion mutant of *M. thermautotrophicus* ΔH contains no Mth60 fimbriae (Fig. 2C and 3C). This loss of Mth60 fimbriae did not appear to influence the generation of individual multicellular filaments. However, the connections to other multicellular filaments were significantly reduced. From this, we concluded that Mth60 fimbriae play a significant role in *M. thermautotrophicus* ΔH biotic cell-cell connections under the conditions we investigated. However, another mechanism seems essential for forming multicellular filaments, which could include a putative influence of additional cell appendages or surface structures. Thus, we argue that Mth60 fimbriae cannot be the only factor essential for forming multicellular filaments, as these multicellular filaments were present in all *M. thermautotrophicus* ΔH strains we analyzed. It was shown that adding Mth60-fimbria antibodies to surface-adhered *M. thermautotrophicus* ΔH cells led to the detachment of the cells, potentially by blocking the Mth60 fimbria adhesion mechanism (20). With the deletion of the Mth60-fimbria operons, and therefore the loss of Mth60 fimbriae, we could now support these results on a genetic level by demonstrating reduced cell-cell connections *in vivo*. Because the raised antibodies against Mth60-fimbriae potentially bound mostly glycosylated proteins, we hypothesized that attachment of the anti-Mth60-fimbria antibody had an effect similar to that of the deletion of oligosaccharyltransferase AglB in *M. maripaludis*. This deletion led to the loss of glycosylation of *M. maripaludis* pilus structures, and therefore the deletion strain was deficient in surface attachment (45). Further investigations of the glycosylation of Mth60 fimbriae will be required in *M. thermautotrophicus* ΔH.

We demonstrated that deletion of all four genes that are cotranscribed with *mth60*, including *mth60*, led to the loss of Mth60 fimbriae. In addition, we provided further evidence for the regulation of the Mth60 fimbria-encoding operons. Based on these findings, the functions of the individual genes in the Mth60 fimbria-encoding operons can be studied in more detail. Understanding the putatively regulated promoters of the Mth60 fimbria-encoding operons is the first step in identifying a sensory system in *M. thermautotrophicus* ΔH that allows adherence to biotic and abiotic surfaces for initial biofilm formation. The reduced ability to form cell-cell connections might impact the rheology of a high-density microbial culture, and thus for biotechnological applications with *M. thermautotrophicus*, such as for power-to-gas processes in large-scale fermentation to convert carbon dioxide and hydrogen to renewable methane (46). A possible effect of the rheology on parameters, such as mixing, gas solubility, and gas conversion efficiency, with the pilus-deficient strain of *M. thermautotrophicus* ΔH will have to be addressed in future research.

## MATERIALS AND METHODS

### Microbial strains, media, and cultivation conditions.

For cloning/gene manipulation and DNA transfer into *M. thermautotrophicus* ΔH, we utilized the Escherichia coli strains NEB stable (New England Biolabs, Frankfurt/Main, Germany) and S17-1 (47), respectively. We cultivated E. coli in LB medium that contained sodium chloride (10 g/L), yeast extract (5 g/L), and tryptone (10 g/L). For solidified LB medium plates, we added 1.5 weight% of Kobe I agar (Carl Roth, Karlsruhe, Germany). We supplemented the LB medium with antibiotic substances for complementary antibiotic resistance genes on plasmids and, when integrated into genomic DNA of E. coli S17-1, with chloramphenicol (30 μg/mL; plasmids) and trimethoprim (10 μg/mL; E. coli S17-1). We incubated all E. coli cultures at 37°C. We incubated solidified medium plates upside down in a static incubator and liquid cultures in a shaker incubator with rotation (150 rpm).

For genetic modification and phenotypical analysis, we purchased *M. thermautotrophicus* ΔH (DSM 1053) from the German Collection of Microorganisms and Cell Cultures (Deutsche Sammlung von Mikroorganismen und Zellkulturen [DSMZ], Braunschweig, Germany). We cultivated *M. thermautotrophicus* ΔH in mineral medium that contained sodium chloride (0.45 g/L), sodium hydrogen carbonate (6.00 g/L), di-potassium hydrogen phosphate (0.17 g/L), potassium di-hydrogen phosphate (0.23 g/L), ammonium chloride (0.19 g/L), magnesium chloride hexahydrate (0.08 g/L), calcium chloride dihydrate (0.06 g/L), ammonium nickel sulfate (1 mL; 0.2 weight%), iron(II)chloride pentahydrate (1 mL; 0.2 weight%), resazurin indicator solution (4 mL; 0.025 weight%), and trace element solution (1 mL; 10-fold as stated in Balch et al. [48]). All chemicals were per analysis (p.a.) grade. We did not add vitamins. For solidified medium plates, we added 1.5 weight% Bacto agar (BD Life Science, Berkshire, UK) prior to autoclaving. Neomycin sodium salt was supplemented for the cultivation of genetically modified *M. thermautotrophicus* ΔH strains with concentrations of 100 μg/mL (initial enrichment step) or 250 μg/mL (all subsequent cultivations) in liquid mineral medium and 100 μg/mL in solidified medium plates at 60°C.

We performed medium preparation based on anaerobic techniques as stated in Balch et al. (48) with the modifications described in Fink et al. (32). In brief, the composed medium was sparged with N_2_/CO_2_ (80/20 volume%). Afterward, for liquid medium, we reduced the medium with 0.5 g/L cysteine hydrochloride, dispensed the medium in serum bottles with a liquid/headspace ratio of 20 mL/80 mL (vol/vol) in an anaerobic chamber with a 100% N_2_ atmosphere (UniLab Pro Eco, MBraun, Garching, Germany), and performed a gas exchange to H_2_/CO_2_ (80/20 volume%). For solidified medium plates, in addition to cysteine hydrochloride, we added 0.3 g/L sodium sulfide monohydrate and dispensed 80 mL into 100-mL serum bottles. We exchanged the gas phase to N_2_/CO_2_ (80/20 volume%) and boiled the medium to liquefy it directly prior to use. An amount of 80 mL per serum bottle was sufficient for 3 to 4 solidified medium plates. We dried the plates for 2 h in the anaerobic chamber. Afterward, the *M. thermautotrophicus* ΔH cell suspension could be plated on the surface (32). We incubated solidified medium plates in pressurized stainless-steel jars (Raff und Grund, Freiberg, Germany) with an H_2_/CO_2_ (80/20 volume%) headspace at 60°C. *M. thermautotrophicus* ΔH in liquid culture was incubated with rotating at 150 rpm at 60°C.

### Molecular cloning and vector construction.

All primers and plasmids from this study are given in Tables 1 and 2. We used a gBlock (gBlock_P*_hmtB_*_P*ac*I_*mth61*; IDT, Coralville, IA, USA) to exchange the native to the P*_hmtB_* promoter. (CGGCTCTAGCTATGTCCGATCAATCTTAATTAAGCCTGGAGGAATGCCCCCATGAACCAACCGATGGCTCAGAAAAACCTTAAAATTAGCGATATATTTATATAGGATTATATGAATAGATAATATCACATAAAATGAGGTGGTTAATTATGAGGACGACAGTTATTTCCGTGATTTTATTGTTTCTAATC). We generated the template genomic DNA from *M. thermautotrophicus* ΔH for PCR amplification using a genomic DNA (gDNA) extraction kit (Macherey-Nagel, Düren, Germany) with slight modifications. Instead of bead beating, we vortexed the cell suspension for 1 min at 4-s intervals and eluted genomic DNA in 50 μL water instead of 100 μL elution buffer. We performed PCR amplification of vector and insert fragments with Q5 high-fidelity polymerase (New England Biolabs, Frankfurt/Main, Germany), followed by DpnI restriction enzyme digestion when necessary. We purified all PCR products via a PCR purification kit (Qiagen, Hilden, Germany) and extracted vector DNA from E. coli via a QIAprep Spin miniprep kit (Qiagen) prior to restriction enzyme digestion. Afterward, we performed the restriction enzyme digestion and fragment ligation according to the manufacturer’s manual. We purchased all enzymes from New England Biolabs. We (re)transformed E. coli with vector constructs by chemical transformation following a standard heat shock protocol (49). We confirmed all plasmids and vectors by Sanger sequencing performed at Genewiz (Azenta Life Sciences, Griesheim, Germany).

**TABLE 1:** Primer list

Name	Purpose	Sequence	Reference or source
Seq_CF1	Analysis genome integration Δ*mth*58-61	CCACCAGTTCGACTCCCTGG	32
Seq_CF2	Analysis genome integration Δ*mth*58-61	CTGTTAAAGGCGGGGGTGG	32
Seq_CF3	Analysis genome integration Δ*mth*58-61	CTTGGGTGATGATGGGATGTATTG	32
Seq_CF4	Analysis genome integration Δ*mth*58-61	CGAGGAGAAACACATCCAGCTG	32
Seq_CF5	Analysis pMVS1111A: P*_hmtB_*-*mth*58-61	GTTAATCCAGCACATCCTCC	32
Seq_CF6	Analysis pMVS1111A: P*_hmtB_*-*mth*58-61	CCTGTCCAACTTATACCTTTGG	32
Gib_CF15	Cloning pCF702	CGGCTCTAGCTATGTCCGATC	32
M13 FW	Cloning pCF702	TGTAAAACGACGGCCAGT	Eurofins genomics (Konstanz, Germany) standard primer list
Res_CF3	Cloning pCF702	TAGCCTATTCGGCGCGCCGAAATCCCAACCTTCATATAATATTGCAAG	This study
Res_CF4	Cloning pCF702	TCATGGCTACGTCGACCGGGCCATAACACATACCAC	This study
Res_CF5	Cloning pCF702	TCAGGATTCGGGCCGGCCTCCGTAAGAAGGGAATTGAACCTCC	This study
Res_CF6	Cloning pCF702	CGTTGCAATGCATATGGGTACTGTGTGAGGGTCATATTCTG	This study
Res_CF7	Cloning pCF702	TATTGCAATGGTCGACAGTGGGCAAGTTGAAAAATTCAC	This study
Res_CF8	Cloning pMVS1111A: P*_hmtB_*-*mth*58-61	ATGCCATTACGGCGCGCCTCACCACACTGTGGCTCCG	This study
Res_CF9	Cloning pCF702	TACGTTGCCAGGCCGGCCCCCATGAACCAACCGATGGC	this study
Res_CF10	Cloning pMVS1111A: P*_hmtB_*-*mth*58-61	CGTTGATCCGTTAATTAAGTCCAGGAATAAGGAAGACATCCCG	This study
Seq_CF9	Analysis genome integration Δ*mth*58-61	GTGGTCCGCTGGCAGATATG	This study
Seq_CF10	Analysis genome integration Δ*mth*58-61	GCTGCTTCCCTGAGGATGTC	This study
Seq_CF11	Analysis genome integration Δ*mth*58-61	CTGCTCCCTGGGATGCCTTG	This study
Seq_CF12	Analysis genome integration Δ*mth*58-61	GTAATCCCGCTTATGGTTGCCC	This study

**TABLE 2:** Plasmid list

Name	Function	Reference or source
pMTL83151	Shuttle vector for *Clostridia* spp.	50
pSB1	Exchange of P*_mcrB_*_(_*_M.v_*_.)_ promoter to P_synth_ for Neo^r^	32
pCF204	pUC57 vector, including Neo^r^ controlled by P*_mcrB_*_(_*_M.v_*_.)_	32
pMSV1111A: P*_hmtB_*-*bgaB*	Shuttle vector construct, including β-galactosidase (*bgaB*)-gene and promoter P*_hmtB_*	32
pCF702	Flanking regions of *mth58-61* with neomycin resistance in between controlled by P_synth_	This study
pMVS1111A: PhmtB-*mth58-61*	*mth58*-*mth61* operon from *M. thermautotrophicus* ΔH controlled by P*_hmtB_* promoter	This study

We generated a shuttle-vector construct for the constitutive expression of the Mth60-fimbria operons. For this, we PCR amplified the genes *mth58* to *mth61* (MTH_RS00275 to MTH_RS00290) without the putative native promoter region upstream of *mth61* with primers Res_CF8 and Res_CF10 (Table 1). Afterward, we fused this PCR product with a gBlock containing the P*_hmtB_* promoter via overlap extension PCR. This PCR amplicon contained the modular restriction sites PacI and AscI and could, thus, be fused after restriction enzyme digestion to the PacI and AscI digested pMVS1111A:P*_hmtB_*-*bgaB*, resulting in pMVS1111A:P*_hmtB_*-*mth58-61* (Table 2).

To generate suitable suicide-vector constructs for genome integration of a thermostable neomycin resistance gene (Neo^r^) (32) at the Mth60 fimbria-encoding operon site, we deployed a three-step cloning strategy (File S1). In brief, the E. coli backbone was fused, first, with 1-kb upstream and, second, with 1-kb downstream homologous flanking regions of the Mth60 fimbria-encoding operons flanking a construct containing Neo^r^ and P*_mcrB_*_(_*_M.v_*_.)_ as a nonfunctional spacer. In the third step, we exchanged the spacer flanked by FseI and AscI as a modular restriction enzyme recognition site toward the functional selectable marker module Neo^r^ with P_Synth_ in further suicide-vector constructs (32, 50).

### Conjugation of *M. thermautotrophicus* ΔH.

We performed conjugations of *M. thermautotrophicus* ΔH with an interdomain conjugation protocol for DNA transfer from E. coli S17-1 to *M. thermautotrophicus* ΔH as described in Fink, et al. (32). In short, we centrifuged 10 mL of stationary E. coli S17-1 that contained the shuttle- or suicide-vector construct at 3,700 rpm for 10 min at room temperature (centrifuge 5920 R, rotor S-4 × 1000, Eppendorf, Hamburg, Germany). We mixed the E. coli S17-1 cell pellet with a cell pellet from 8 mL of a stationary-grown *M. thermautotrophicus* ΔH culture that we stepwise harvested inside the anaerobic chamber at 12,500 rpm for 4 min at room temperature (MySPIN 12 mini centrifuge, Thermo Scientific, Waltham MA, USA). Afterward, the cell suspension of E. coli and *M. thermautotrophicus* ΔH was spot-mated on a solidified medium plate containing 50 volume% LB medium without sodium chloride and 50 volume% mineral medium. After the cell suspension was completely absorbed, the plate was incubated for 24 h at 37°C in a pressurized stainless-steel jar with an H_2_/CO_2_ (80/20 volume%) headspace. The incubated spot-mated cells were washed off the plate, transferred into sterile anaerobic mineral medium, and incubated for 4 h at 60°C for recovery, expression of the neomycin resistance gene, and counterselection against E. coli. After the recovery, the *M. thermautotrophicus* ΔH mutants were enriched in 100 μg/mL (suicide vector) or 250 μg/mL (shuttle vector) neomycin-containing selective liquid medium. The stationary-grown enrichment culture was spread-plated on selective solidified medium plates, and individual clonal populations were subjected to further analysis via PCR.

### Confirmation of *M. thermautotrophicus* ΔH mutant strains via PCR analysis.

For screening purposes, we resuspended an individual clonal population in 50 μL of nuclease-free water or used 0.1 mL of *M. thermautotrophicus* ΔH culture directly and boiled the suspension at 100°C for 12 min prior to using 1 μL of suspension for PCR analysis. A final analysis was performed with 1 μL of genomic DNA extractions of the *M. thermautotrophicus* ΔH mutant strains as the template DNA for 10-μL PCR mixes. PCR analysis was performed using the Phire plant PCR master mix (Thermo Scientific, Waltham, MA, USA). The denaturation and annealing times were increased to 20 s and to 10 s, respectively. A total of 30 cycles were performed for all analyses. We observed false-positive PCR signals for shuttle-vector DNA and suicide-vector constructs due to plasmid DNA carryover from E. coli for up to two transfers after the nonselective liquid recovery step. From the third transfer, plasmid DNA from E. coli was no longer detectable in any of our experiments.

### Immunofluorescence staining.

For immunofluorescence staining analysis, we placed 20 μL of late-exponential-phase *M. thermautotrophicus* ΔH culture on a poly-l-lysine-coated glass slide (VWR, Darmstadt, Germany). After allowing cells to settle onto the glass slide for 20 min, we washed the glass slide three times, for 5 min each, with phosphate-buffered saline (PBS, pH 7.4). Afterward, we applied the anti-Mth60-fimbria antibody (1:2,000 diluted; rabbit) (20) in PBS, containing 0.3 weight% bovine serum albumin (BSA; Carl Roth, Karlsruhe, Germany) and incubated the mixture for 2 h. We washed the sample three times, for 5 min each, with PBS (pH 7.4). Then, we applied a goat anti-rabbit IgG (Thermo Fisher Scientific, Waltham, MA, USA) cross-adsorbed secondary antibody with Alexa Fluor 488 (diluted 1:2,000) in PBS containing 0.3 weight% BSA and incubated it for 1 h. The incubation was followed by three additional washing steps with PBS (pH 7.4) to reduce the background. After the sample was almost dry, we applied 10 μL of Invitrogen ProLong Gold antifade mountant with DAPI (4′,6-diamidino-2-phenylindole) and covered it with a cover glass. Prolong Gold antifade mountant was allowed to solidify at 4°C for 24 h prior to Airyscan imaging analysis.

We performed Airyscan imaging at the Max Planck Institute for Biology Tübingen BioOptics Facility using a laser scanning inverted confocal microscope (LSM 780; Carl Zeiss AG, Oberkochen, Germany) with a 63× oil/1.4 numerical aperture (NA) oil-immersion objective. We used the 405-nm diode laser line for the excitation of DAPI and the 488-nm Argon laser line for the excitation of Alexa Fluor 488-conjugated antibody.

For the Airyscan images, we used the add-on Airyscan detection unit (Carl Zeiss AG), set to superresolution mode (SR). For each area, we acquired a z-stack of images and processed the obtained data set first through the Airyscan software, which operates a 3D deconvolution on top of the pixel reassignment (51), and then by maximum intensity projection along the *z* axis to easily visualize the collected information on a single plane.

### Scanning electron microscopy.

For scanning electron microscopy (SEM) analysis, we coated glow-discharged glass slides with 30 μL of 0.1% poly-l-lysine (Plano, Wetzlar, Germany, item number 18026) and dried them in a 60°C incubator oven for 1 h. In a fume hood, we placed a prepared glass slide (coated-side up) at the bottom of each well of a 24-well plate. We separately added a 100-μL aliquot of each strain (i.e., late-exponential-phase culture of *M. thermautotrophicus* ΔH wild-type, constitutive expression, and deletion strains) to a well, and cells were allowed to settle onto the glass slide. After 20 min of incubation, we removed the supernatant and added 100-μL PBS. Electron microscopy-grade glutaraldehyde (25%, Plano, Agar Scientific, item number R1011) was added to each sample to obtain an overall 2.5 volume%. Afterward, we covered and incubated the 24-well plate at room temperature (ca. 21°C) for 1 h, allowing for fixation and for cells to settle onto the slides. After incubation, we removed the supernatant from each well and rinsed the sample-bearing slides by adding deionized water to each well and incubating them for 10 min to remove material that did not attach to the glass slide. This rinsing procedure was repeated by removing the supernatant and adding fresh deionized water. After the final rinse, we dehydrated the samples using a graded ethanol series: 25, 50, and 75 volume% ethanol (15-min incubation at each concentration) and three times 100 volume% ethanol (30-min incubations). After the last ethanol dehydration, we added 100 volume% hexamethyldisilazane (HMDS) to each sample so that each well contained a 50/50 volume% ratio of HMDS/100 volume% ethanol. Then, we covered the plate and allowed it to incubate for 30 min. After incubation, we removed the HMDS/ethanol solution and added 100 volume% HMDS to each well. We left the plate lid partially open to allow air-drying to occur overnight. The sample-bearing glass slides were adhered to aluminum stubs using carbon adhesive tabs (Plano, item numbers G301 and G3347) and coated them with ca. 8 nm of platinum using a BAL-TEC SCD 005 sputter coater. We performed the structural characterization of *M. thermautotrophicus* ΔH strains using a Zeiss Crossbeam 550L focused ion beam (FIB) scanning electron microscope (Oberkochen, Germany), operating with an acceleration voltage of 2 kV. We took all micrographs using secondary electron (SE) mode.

### Phase-contrast microscopy analysis.

We placed 10 μL of untreated late-exponential-phase (OD_600_, 0.28) *M. thermautotrophicus* ΔH cultures (each of wild-type, constitutive expression, and deletion strains) on microscopy slides and added coverslips (*n* = 3 for each strain). After 10 min of incubation at room temperature, 30 pictures (10 pictures of each biological replicate) were taken for all three *M. thermautotrophicus* ΔH strains. We chose random vision fields at 100-fold magnification and phase contrast 3 with a System Microscope BX41TF (Olympus, Shinjuku, Japan; equipped with a U-TV0.5XC-3 camera).

In all 30 pictures for each *M. thermautotrophicus* ΔH strain, we counted the total number of microbes and the number of microbes connected to another microbe. Afterward, we calculated the ratio between the total number and the number of connected microbes using R (52, 53). Statistical differences among the three investigated strains were analyzed with a single-factor ANOVA. When *F* exceeded critical *F* value, we performed two-sided heteroscedastic Student’s *t* tests.

### Measurement of size distribution.

We measured the size distribution of the *M. thermautotrophicus* ΔH strains with a Zetasizer Nano ZSP (Malvern, Herrenberg, Germany). Therefore, we incubated the strains with rotation (100 rpm) at 60°C as described above. However, we omitted resazurin from the medium to avoid interference with the Zetasizer measurement. Then, 1 mL of late-exponential-phase *M. thermautotrophicus* ΔH cultures (each of wild-type, constitutive expression, and deletion strains) was transferred into a disposable cuvette. Size measurements were performed in biological and technical triplicate. We performed the same statistical approach as for the phase-contrast microscopy analysis.

### Western blot analysis of crude cell extracts.

For Western blot analyses, we harvested 10 mL of late-exponential-phase *M. thermautotrophicus* ΔH cultures (each of wild-type, constitutive expression, and deletion strains) by centrifugation at 13,000 rpm for 2 min at room temperature (mini centrifuge MySPIN 12, 12 × 1.5/2.0-mL tube rotor, Thermo Fisher Scientific, Dreieich, Germany). We resuspended the cell pellets in 500 μL fresh mineral medium and added pseudomurein endoisopeptidase (PeiP), which we produced as described before (54), at 20 μL/500 μL cell suspension for cell lysis. After incubation at 60°C for 30 min, we separated the cell-free extract (supernatant) from the insoluble fraction by centrifugation at 13,000 rpm for 2 min at room temperature (mini centrifuge MySPIN 12, 12 × 1.5/2.0 mL tube rotor, Thermo Fisher Scientific). The fractions were prepared for SDS-PAGE by adding 4× SDS-sample buffer (100 mM Tris-HCl, pH 6.8, 40 volume% glycerol, 110 mM SDS, 2 mM β-mercaptoethanol, 3 mM bromophenol blue) and heating at 95°C for 15 min and centrifugation at 13,000 rpm for 2 min at room temperature. Samples were stored at −20°C until use. For the SDS-PAGE, 10 μL of each fraction from each strain was loaded onto 15% polyacrylamide gels and separated for 45 min at 45 mA per gel (EasyPhor PAGE mini vertical electrophoresis chamber, Biozym Scientific GmbH, Hessisch Oldendorf, Germany). We replicated the gels to stain one gel with InstantBlue protein stain (Expedeon Ltd., Cambridge, UK) according to the manufacturer’s recommendations and a second gel for Western blot analysis. For Western blot analysis, the proteins from the gel were transferred to a methanol-activated polyvinylidene difluoride (PVDF) membrane (Immobilon-P PVDF membrane, 0.45 μm, Merck Millipore Ltd., Merck KGaA, Darmstadt, Germany), which was incubated for 5 to 10 min in blotting-transfer buffer (25 mM Tris-HCl, pH 8.5, 150 mM glycine, 10 volume% methanol). We performed semidry blotting in a Trans-Blot Turbo transfer system (Bio-Rad Laboratories GmbH, Feldkirchen, Germany) for 30 min at 10 V. Afterward, we treated the membrane according to a protocol for the chromogenic detection with alkaline phosphatase (“Strep-tag detection in Western blots,” IBA GmbH, Göttingen, Germany) with slight modifications. In brief, the membrane was blocked in 20 mL Tris-buffered saline (TBS) blocking buffer (20 mL TBS buffer, 3 weight% BSA, 0.1 volume% Tween 20) for 1 h followed by three 5-min washing steps in 20 mL TBS-Tween buffer (20 mL TBS buffer, 0.1 volume% Tween 20) with gentle shaking. Then, the membrane was incubated with the anti-Mth60-fimbriae antibody as the primary antibody (1:5,000, rabbit-raised) in 10 mL TBS-Tween buffer for 1 h at room temperature. Nonbinding antibodies were washed away by washing twice, first with 20 mL TBS-Tween buffer and then with 20 mL TBS buffer. For a chromogenic reaction treatment via alkaline phosphatase (AP), the membrane was incubated in 10 mL TBS-Tween with a secondary antibody conjugated with AP (goat anti-rabbit IgG (H+L), AP; Thermo Fisher Scientific) (1:5,000) for 1 h at room temperature, followed by a 20-mL TBS-Tween and 20-mL TBS washing step repeated twice. The membrane was transferred to 20 mL reaction buffer (100 mM Tris-HCl, pH 8.8, 100 mM NaCl, 5 mM MgCl_2_, 10 μL 7.5 weight% nitrotetrazolium blue solution, 60 μL 5 weight% 5-bromo-4-chloro-3-indolyl phosphate solution). After 20 min of development, the AP reaction was visible directly on the membrane by a colored signal.

### Data availability.

The Oxford Nanopore sequencing raw data are available at NCBI under BioProject ID PRJNA967643.
